# Cross-validation-based sequential design for stochastic models

**DOI:** 10.1098/rsta.2024.0217

**Published:** 2025-04-02

**Authors:** Louise M. Kimpton, Michael Dunne, James M. Salter, Peter Challenor

**Affiliations:** ^1^ Department of Mathematics and Statistics, University of Exeter, Exeter, UK

**Keywords:** stochastic computer models, Gaussian process emulators, sequential design, exploration versus replication, leave-one-out cross-validation

## Abstract

Complex numerical models are increasingly being used in healthcare and epidemiology. To represent the complex features, modellers often make the decision to include stochastic behaviour where repeated runs of the model with identical inputs produce different outputs. When computational constraints limit the number of model runs and replications, heteroscedastic Gaussian processes can be used as a fast surrogate, allowing for efficient emulation of varying noise levels across the input space. The accuracy of any emulator is greatly dependent on the design of the training data, where sequential design algorithms increase the number of design points iteratively based on predefined criteria. For stochastic models, the design problem is more challenging due to the possibility of replicates at design points. This article develops a new sequential design method for stochastic models which scales well in high-dimensional input spaces. We build upon an existing method for deterministic models using an expected squared leave-one-out error criterion that balances exploration and replication. We compare our approach with existing sequential design methods as well as applying it to an agent-based model and a COVID-19 model. Results demonstrate that the proposed method performs well in noisy environments, offering a scalable alternative to existing methods.

This article is part of the theme issue ‘Uncertainty quantification for healthcare and biological systems (Part 2)’.

## 1. Introduction

Complex numerical models are used to describe and represent real-world processes in engineering and physical science applications [1]. From the success seen in these applications, there has been a drive to increase the use of simulators and digital twins in both healthcare and biological applications [2,3]. Unlike physical and engineering problems, where typically the relationship between inputs and outputs is deterministic, in biology and epidemiology [4], the models tend to be stochastic: every time the same input parameters are passed through the complex model, different outputs are produced. Biological applications often require the presence of random elements (as a modelling choice) to best represent highly complex and uncertain physiologies. For example, agent-based models (ABMs), which are becoming more common in social science and biology, tend to involve many agents that randomly evolve and interact with other agents, causing potentially high levels of stochasticity. The variance of this increased noise is known as the intrinsic variance and is typically dependent on the input parameters themselves, and is important to accurately quantify.

If the computer model is quick, and hence cheap to run, we are able to both fully explore the input space as well as run sufficient numbers of replicates. Running replicates of the model at the same input parameters is valuable for stochastic simulators to gain knowledge about the intrinsic variance. With the increase in complexity of computer models, this is not always possible and we may be very limited in our choice of model runs and possible replications. We turn to emulators, in particular Gaussian processes (GPs) [5–7], which are able to efficiently approximate the full complex model while quantifying the additional level of uncertainty.

For any GP (or emulator), the fit and accuracy are greatly dependent on the design of the training data [1,6,8,9]. It is important to carefully balance between choosing a space-filling design and one that captures the important features of the physical system [10]. This is known as the trade-off between exploration and exploitation [11–13]. Exploration focuses on sampling areas with high uncertainty to improve the global fit of the emulator. This typically leads to a space-filling design, placing points in previously unexplored areas of space. Alternatively, exploitation samples local regions of the input space that are considered interesting and need further points to reduce the emulator uncertainty in these areas. The underlying function in such regions may contain maxima, abrupt changes or be highly nonlinear. Optimum designs are divided into two categories: single-shot methods where the entire budget is used at once, or adaptive methods, where new parameter locations are chosen sequentially based on some predefined criteria. Methods include Latin hypercubes [14], maximum entropy [15], minimum predictive uncertainty [1], mutual information [16] and expected improvement (EI) [17]. Adaptive methods are particularly useful since new design points are chosen one (or more) at a time using information from the emulator and the existing design.

The difference between choosing a design for stochastic models compared with deterministic models is the use of replicates. A simple approach for a single-shot method is to select an optimum space-filling design, such as a Latin hypercube, and then determine the number of replicates to perform at each selected design point. This is typically selected based on the available total budget, see [18] and [19].

Sequential designs are challenging for stochastic models since at each iteration, one has the choice to either add a new point to the design, or to repeat an existing point. While including a new point should increase the accuracy of predicting the mean response, further replicating existing design points should give valuable information regarding the intrinsic variance. As a naive approach, it is possible to use a deterministic sequential design to select new points, while hand picking when and where to include replicates [20–24]. However, this does not sequentially optimize each run of the computer model. Chen & Zhou [25], improve on this to carefully consider the balance between the number of new points and the number of replications.

Binois *et al*. [26] developed a solution to the stochastic sequential design problem using integrated mean squared prediction error (IMSPE) combined with a widely known heteroscedastic (varied noise levels across the input space) Gaussian process method known as hetGP [27]. HetGP assumes the intrinsic variances at the design points to be unknown parameters and reduces the computational complexity of estimating these parameters by using Woodbury matrix identities to reduce the dimension of the likelihood. The IMSPE sequential design methodology starts with a space-filling design ${\text{X}}_{0}$ and an initial allocation of replicates at each point. A GP is then fitted to this design using hetGP and an estimate of the IMSPE of this design is estimated as $\text{IMSPE}({\text{X}}_{0})$ . At each iteration, an input location x is chosen either as a new point or a replicate if it minimizes $\text{IMSPE}({\text{X}}_{0}\cup \text{x})$ . As an extension, they also propose a ’looking ahead over replication’ step that evaluates different decision paths to determine when it is best to either explore space or replicate. For example, if a new point is chosen, then this location is considered as a candidate for future replication. The IMSPE is calculated for each possible path up to a certain number of steps in the future and the potential path with the smallest IMSPE is chosen. There are very few other options for heteroscedastic sequential design methods, which include [28] and [29] in the Bayesian optimization literature, however hetGP remains the leading method.

Although the IMSPE method has proven effective for stochastic models, it is no longer the leading method when it comes to deterministic models [30]. One significant issue is its scalability in higher dimensions. Optimizing IMSPE globally in high dimensions can lead to over-sampling regions with high predictive uncertainty. These areas of high uncertainty can typically be located in the edges of the input space where the model is often not of interest and additional points do not contribute to the increased accuracy of the emulator. IMSPE tends to prioritize reducing uncertainty without considering the broader design objectives, such as exploring areas of interest with specific response surface features. The focus of this article is to develop a new sequential design method suitable for noisy biological models that also scales well in higher dimensions. In particular, models in epidemiology tend to have very intricate variation due to the complex random nature of the components. While IMSPE remains effective in many scenarios, its limitations, particularly in high-dimensional spaces and complex noise structures, highlight the importance of exploring alternatives.

We adapt the expected squared leave-one-out (ES-LOO) approach from Mohammadi *et al*. [31]. The authors generated a new adaptive design criterion for deterministic models based on calculating a leave-one-out cross-validation (LOO-CV). They first build a GP on an initial design and calculate the expected squared LOO-CV error. To apply this over the whole input space they model the LOO-CV error as a GP and find the maximum using a variation of EI [31]. First, we incorporate the hetGP method [27] to build heteroscedastic GPs. Second, to ensure that we balance the relationship between exploration and replication, we select both a new point and replicate simultaneously based on adaptations of the ES-LOO criterion, and select the optimum choice between these candidates. This is similar to van Beek *et al*. [32], who select between a new point and a replicate using maximum predictive variance. We have chosen this method first because it is computationally simple and easy to implement. It has also been shown to be successful in high dimensions, and has the option of choosing a new point through optimization instead of a candidate set. To test the suitability of the new design approach in the context of an epidemiological model with high intrinsic variance and high dimension we use an ABM infectious disease model as well as a COVID-19 model that simulates the spread of the virus in Scotland [33].

### (a) Heteroscedastic Gaussian processes for stochastic models

Let the complex computer model be represented by the function f:X↦ℝ with $\text{X}=\left({\text{x}}_{1},\ldots ,{\text{x}}_{N}\right)$ being a set of N inputs to the model, each of which has p dimensions with ${\text{x}}_{i}\in X$ . The function f(⋅) maps the inputs X to their outputs $\text{y}=(f\;({\text{x}}_{1}),\ldots ,f\;({\text{x}}_{N}){)}^{T}$ , and can be represented by:


(1.1) $$f(\text{x})=F(\text{x})+v\;\text{with }v∼N(0,\tau^{2}(\text{x})).$$

For deterministic simulators, $\tau^{2}=0$ , while for stochastic models, $\tau^{2}$ is the intrinsic variance and can either be constant or depend on the inputs x . We define F(x)=E(f(x)) as the expected value of the output. We can model f as a Gaussian process Z with training data D={X,y} such that


(1.2) $$\begin{array}{lcr} & Z(\text{x})∼GP(m(\text{x}),k(\text{x},{\text{x}}^{\prime })+\tau^{2}(\text{x})), & \end{array}$$

where m(⋅) and k(⋅,⋅) are predefined mean and covariance functions. Using standard multi-variate Normal distribution results, we can predict the function f at a new location x with posterior mean and variance given as:


(1.3) $$\begin{array}{lcr} & \begin{array}{rl} m^{*}(\text{x}) & =m(\text{x})+k(\text{x},\text{X})(K_{N}+\tau^{2}(\text{x})I_{N}{)}^{-1}(\text{y}-m(\text{X})),\\ k^{*}(\text{x}) & =\tau^{2}(\text{x})+k(\text{x},\text{x})-k(\text{x},\text{X})(K_{N}+\tau^{2}(\text{x})I_{N}{)}^{-1}k(\text{X},\text{x})\\ & =\tau^{2}(\text{x})+k_{Z}(\text{x}), \end{array} & \end{array}$$

where k(x,X) is an *N*-vector whose $i^{\text{th}}$ component is $k(\text{x},{\text{x}}_{i})$ for i=1,…,N , $K_{N}=k(\text{X},\text{X})$ is an N×N matrix with entries $k_{ij}=k({\text{x}}_{i},{\text{x}}_{j})$ and $I_{N}$ is the N×N identity matrix. A common choice for k are Matérn covariance functions defined as follows:


(1.4) $$k(\text{x},{\text{x}}^{\prime })=\sigma^{2}\frac{2^{1-\nu }}{\Gamma (\nu )}(\frac{\sqrt{2\nu }}{\theta }|\text{x}-{\text{x}}^{\prime }|{)}^{\nu }B_{\nu }(\frac{\sqrt{2\nu }}{\theta }|\text{x}-{\text{x}}^{\prime }|),$$

where Γ(⋅) is the Gamma function and $B_{\nu }(\cdot )$ is a modified Bessel function of the second kind with order ν . Here, ν controls the smoothness of the resulting GP and $\sigma^{2}$ and θ are the variance and correlation length parameters, respectively.

If we have replicated runs of the model at some of the design points, a common approach in the literature is to fit an independent GP to $\tau^{2}(\text{x})$ [20,34,35]. Let ${\text{X}}^{\prime }=\left\{{\text{x}}_{1}^{\prime },\ldots ,{\text{x}}_{n}^{\prime }\right\}$ for n≪N represent the unique inputs within X . If there are enough replicates at each of the design points, we can approximate both F and $\tau^{2}$ by calculating the sample means and sample variances:


(1.5) $$\begin{array}{lcr} & \begin{array}{rl} \bar{y}({\text{x}}_{i}^{\prime }) & =\frac{1}{r_{i}}\sum_{j=1}^{r_{i}}f({\text{x}}_{ij}),\\ s^{2}({\text{x}}_{i}^{\prime }) & =\frac{1}{r_{i}-1}\sum_{j=1}^{r_{i}}(f({\text{x}}_{ij})-\bar{y}({\text{x}}_{i}^{\prime }){)}^{2}, \end{array} & \end{array}$$

where $r_{i}$ is the number of replicates at ${\text{x}}_{i}^{\prime }$ for i=1,…,n , and where j are the indices representing the replicates at each ${\text{x}}_{i}^{\prime }$ . Hence, $f({\text{x}}_{ij})$ is the $j^{\text{th}}$ replicated output from running the model at input ${\text{x}}_{i}^{\prime }$ . Homoscedastic GPs can then be used for the mean and log of the variance [20,35–37]. When there are very few replicates, the sample statistics for the mean and variance are not good estimates for the true mean and heteroscedasticity.

To solve this problem, methods such as [35–37] consider the intrinsic variances $\tau^{2}$ to be latent parameters. A computationally efficient approach was developed by Binois *et al.* [27], known as hetGP. First define the latent variables to be $\Lambda_{n}=(\lambda ({\text{x}}_{1}^{\prime }),\ldots ,\lambda ({\text{x}}_{n}^{\prime }))$ with $\lambda (\text{x})=\frac{\tau^{2}(\text{x})}{\sigma^{2}}$ , where $\sigma^{2}=k(\text{x},\text{x})$ is the variance parameter of the GP. The goal is to learn the values in $\Lambda_{n}$ , which is expensive if done via Markov Chain Monte Carlo (MCMC). They solve this issue by modelling the log variances as the mean output of a GP on latent variables as well as using Woodbury identities to reduce the number of latent variables and replicates at each design point are not required.

The authors assume a GP prior for logλ that constrains $\text{log}\Lambda_{n}$ to have a multi-variate Normal distribution. Following this, they state that $\lambda_{1},\ldots ,\lambda_{n}$ from $\Lambda_{n}$ become quantities from the predictive mean of a GP trained on new latent variables $\Delta_{n}=\text{diag}(\delta_{1},\ldots ,\delta_{n})$ . If the covariance function of $\Delta_{n}$ is $C_{\left(g\right)}(C_{\left(g\right)}+gR^{-1})$ with $R_{n}=\text{diag}(r_{1},\ldots ,r_{n})$ , then assuming the GP has zero mean, the following holds:


(1.6) $$\text{log}\Lambda_{n}=C_{\left(g\right)}(C_{\left(g\right)}+gR_{n}^{-1}{)}^{-1}\Delta_{n},$$

where $C_{\left(g\right)}$ with $K_{n}+\tau^{2}(\text{x})I_{n}=\sigma^{2}(C_{n}+\Lambda_{n})$ is a correlation function with nugget parameter g . This approach enables smooth estimation of $\Lambda_{n}$ and provides a fixed functional form for λ(x) . However, it does not account for the uncertainty introduced by the estimation of the intrinsic variance $\tau^{2}(\text{x})$ when making predictions. The estimates for $\tau^{2}(\text{x})$ are assumed without error and so no uncertainty is included in predictions. With $\Lambda_{n}$ specified, the Woodbury identities simplify the likelihood computation for y which includes all outputs and replicates, reducing the dependence to quantities of size n . The unknown parameters can then be estimated using maximum likelihood at a computational complexity of $O(n^{3})$ . Full details are given in [27]. We will be using hetGP for heteroscedastic GPs in the rest of the paper.

## 2. Sequential design for stochastic models

Our approach for heteroscedastic sequential design is based on a deterministic version introduced by Mohammadi *et al*. [31], which combines the ES-LOO and pseudo-expected improvement (PEI) to ensure a suitable trade-off between local exploitation and global exploration of the design. For deterministic models, the ES-LOO methodology chooses the point that maximizes the PEI. For stochastic models, we have the additional choice to either choose the new point, or to perform a replication of an existing point in the design.

Running the model at a new point allows for both an improved estimate of the mean response as well as better exploration of the input space. This can help to discover regions with high variability or unexpected behaviour, leading to a more comprehensive understanding. Introducing several new points close together can improve estimates of the intrinsic variance since information is shared through the covariance. Replication of existing inputs allows for a better estimate of both the mean and variance at that point, which can help in reducing the impact of stochastic noise and improving the accuracy of the model in a local region.

We choose an approach that carefully considers selecting both an optimum new point as well as an optimum replicate. These selections are made independently using variations of the ES-LOO criteria. We then apply a second criterion, denoted G , to select between running the model at the new point or at the replicate. Given an existing set of design points $\text{X}=\left\{{\text{x}}_{1},\ldots ,{\text{x}}_{N}\right\}$ , with unique points given as ${\text{X}}^{\prime }=\left\{{\text{x}}_{1}^{\prime },\ldots ,{\text{x}}_{n}^{\prime }\right\}$ for n≪N , we propose that the next model input parameter ${\text{x}}^{*}$ is chosen according to the following criterion:


(2.1) $$\begin{array}{rl} {\text{x}}^{*}=\underset{\text{x}\in X,\text{x}\in \text{X}}{\arg\max}\;\{ & G({\text{x}}_{m}^{*}),\;\;c\cdot G({\text{x}}_{\tau }^{*})\}\\ {\text{x}}_{m}^{*}=\underset{\text{x}\in X}{\arg\max}\;\{{\text{ESL}}_{m}(\text{x})\}\; & \text{ and }\;{\text{x}}_{\tau }^{*}=\underset{\text{x}\in \text{X}}{\arg\max}\;\{{\text{ESL}}_{\tau }(\text{x})\}, \end{array}$$

where ${\text{ESL}}_{m}$ is the ES-LOO criteria for including a new point and ${\text{ESL}}_{\tau }$ is the ES-LOO criteria for selecting a replicate. Derivations are provided in §2a(i,ii). Choices for G are given in §2b. The parameter c adds flexibility to the design algorithm. If there is a user preference for including either more new points or increasing the number of replicates then an additional weighting can be included to represent these preferences.

To perform a sequential design, one must start with an initial design. We suggest doing this with either a Latin hypercube, Sobol sequence or equivalent one-shot method [10,14]. At this stage, we aim for a space-filling design to obtain a good first estimate of the GP, making sure that we are not missing out on any crucial areas of input space.

### (a) Few replicates

When there are not enough replicates, the sample mean and sample variance are not good estimates for the true mean and variance and hence we use hetGP to fit a GP. See [20] for a discussion on what is defined as ‘enough replicates’. To calculate ${\text{ESL}}_{m}(\text{x})$ and ${\text{ESL}}_{\tau }(\text{x})$ , the deterministic ES-LOO framework [31] must be adapted. Since the model is stochastic, we do not know either the true mean response or the true intrinsic variance to calculate the LOO error. Since the intrinsic variance is assumed to be estimated ‘perfectly’ with no error, this cannot be taken into account when considering the sensitivity of the GP in the ES-LOO calculation. Finally, maximization of ${\text{ESL}}_{\tau }(\text{x})$ is only required over the existing design points and hence an EI step is not required.

#### (i) New point—calculating ${\text{ESL}}_{m}(\text{x})$

To calculate ${\text{ESL}}_{m}(\text{x})$ required for selecting a new design point, we first fit an initial hetGP Z(x) with training data D={X,y} and predicted mean, $m^{*}$ , and variance, $k^{*}=\tau^{2}+k_{Z}$ . A LOO-CV is then performed on Z(x) such that


(2.2) $$Z_{-i}({\text{x}}_{i}^{\prime })∼N(m_{-i}^{*}({\text{x}}_{i}^{\prime }),k_{-i}^{*}({\text{x}}_{i}^{\prime })),$$

where $Z_{-i}(\text{x})$ is defined to be Z(x) with the $i^{\text{th}}$ unique training point removed and $m_{-i}^{*}$ and $k_{-i}^{*}$ define the LOO predicted mean and variance, respectively. We note that this LOO is performed by removing all $r_{i}$ replicates for each unique point ${\text{x}}_{i}^{\prime }$ since we are only focused on the error on the mean response. The computational cost is kept minimal by not re-estimating GP hyperparameters. The parameters from Z(x) are used in $Z_{-i}(\text{x})$ . Equation (2.2) can be calculated efficiently using the methods proposed in [38].

In the deterministic ES-LOO criterion, the LOO error is calculated by comparing the LOO predicted mean with the true model output at each input. We do not know what the true mean response is at the design points, however, we can consider the sample mean to be a reasonable estimate. Even with small sample sizes, the sample mean tends to converge towards the true mean due to the central limit theorem. Although there is additional error included in this estimate, areas of input space where the difference between the LOO estimate and the sample mean is large are still areas that are the most uncertain and require further exploration. We therefore replace the true mean response $f({\text{x}}_{i}^{\prime })$ at each of the points in the training data with the sample mean of the model outputs across all replicates $\bar{y}({\text{x}}_{i}^{\prime })$ . The LOO-CV error can be calculated as:


(2.3) $$\begin{array}{lcr} & e({\text{x}}_{i}^{\prime })=|m_{-i}^{*}({\text{x}}_{i}^{\prime })-\bar{y}({\text{x}}_{i}^{\prime })|. & \end{array}$$

The LOO error does not account for the difference between the true value at ${\text{x}}_{i}^{\prime }$ and the predicted mean when the input point is left out of the initial design. We calculate the ES-LOO, $E_{m}({\text{x}}_{i}^{\prime })$ to account for the sensitivity of the emulator to the design points. We make the assumption that the sample mean at input ${\text{x}}_{i}^{\prime }$ is a normally distributed random variable, ${\bar{Y}}_{i}$ , with expectation $\bar{y}({\text{x}}_{i}^{\prime })$ and variance $\sigma_{m}^{2}({\text{x}}_{i}^{\prime })$ . However, since we do not know the value of $\sigma_{m}^{2}({\text{x}}_{i}^{\prime })$ , we instead substitute in the predicted variance from Z at input ${\text{x}}_{i}^{\prime }$ , giving an estimate of the variance of the sample mean as $k_{Z}({\text{x}}_{i}^{\prime })/r_{i}$ . By standardizing equation (2.2) with the sample mean, we have the following:


(2.4) $$\begin{array}{lcr} & \frac{Z_{-i}({\text{x}}_{i}^{\prime })-{\bar{Y}}_{i}}{\sqrt{k_{-i}^{*}({\text{x}}_{i}^{\prime })+k_{Z}({\text{x}}_{i}^{\prime })/r_{i}}}∼N\left(\frac{m_{-i}^{*}({\text{x}}_{i}^{\prime })-\bar{y}({\text{x}}_{i}^{\prime })}{\sqrt{k_{-i}^{*}({\text{x}}_{i}^{\prime })+k_{Z}({\text{x}}_{i}^{\prime })/r_{i}}},1\right). & \end{array}$$

By taking the square of the left-hand side, this now becomes a random variable with non-central chi-squared distribution given by:


(2.5) $$\begin{array}{lcr} & {\left(\frac{Z_{-i}({\text{x}}_{i}^{\prime })-{\bar{Y}}_{i}}{\sqrt{k_{-i}^{*}({\text{x}}_{i}^{\prime })+k_{Z}({\text{x}}_{i}^{\prime })/r_{i}}}\right)}^{2}∼{\chi^{\prime }}^{2}\left(\kappa =1,\lambda ={\left(\frac{m_{-i}^{*}({\text{x}}_{i}^{\prime })-\bar{y}({\text{x}}_{i}^{\prime })}{\sqrt{k_{-i}^{*}({\text{x}}_{i}^{\prime })+k_{Z}({\text{x}}_{i}^{\prime })/r_{i}}}\right)}^{2}\right), & \end{array}$$

where κ is the degrees of freedom and λ is the non-centrality parameter. The expectation and variance of the squared quantity are subsequently given by:


(2.6) $$\begin{array}{lcr} & \begin{array}{rl} E\left[{\left(\frac{Z_{-i}({\text{x}}_{i}^{\prime })-{\bar{Y}}_{i}}{\sqrt{k_{-i}^{*}({\text{x}}_{i}^{\prime })+k_{Z}({\text{x}}_{i}^{\prime })/r_{i}}}\right)}^{2}\right] & =1+{\left(\frac{m_{-i}^{*}({\text{x}}_{i}^{\prime })-\bar{y}({\text{x}}_{i}^{\prime })}{\sqrt{k_{-i}^{*}({\text{x}}_{i}^{\prime })+k_{Z}({\text{x}}_{i}^{\prime })/r_{i}}}\right)}^{2},\\ \text{Var}\left[{\left(\frac{Z_{-i}({\text{x}}_{i}^{\prime })-{\bar{Y}}_{i}}{\sqrt{k_{-i}^{*}({\text{x}}_{i}^{\prime })+k_{Z}({\text{x}}_{i}^{\prime })/r_{i}}}\right)}^{2}\right] & =2\left(1+2{\left(\frac{m_{-i}^{*}({\text{x}}_{i}^{\prime })-\bar{y}({\text{x}}_{i}^{\prime })}{\sqrt{k_{-i}^{*}({\text{x}}_{i}^{\prime })+k_{Z}({\text{x}}_{i}^{\prime })/r_{i}}}\right)}^{2}\right). \end{array} & \end{array}$$

Expanding these equations produces the final form of the ES-LOO:


(2.7) $$\begin{array}{rl} E_{m}({\text{x}}_{i}) & =\frac{E[(Z_{-i}({\text{x}}_{i}^{\prime })-\bar{y}({\text{x}}_{i}^{\prime }){)}^{2}]}{\sqrt{\text{Var}[(Z_{-i}({\text{x}}_{i}^{\prime })-\bar{y}({\text{x}}_{i}^{\prime }){)}^{2}]}},\\ \text{where}\; & \\ E[(Z_{-i}({\text{x}}_{i}^{\prime })-\bar{y}({\text{x}}_{i}^{\prime }){)}^{2}] & =k_{-i}^{*}({\text{x}}_{i}^{\prime })+k_{Z}({\text{x}}_{i}^{\prime })/r_{i}+(m_{-i}^{*}({\text{x}}_{i}^{\prime })-\bar{y}({\text{x}}_{i}^{\prime }){)}^{2}\\ \text{Var}[(Z_{-i}({\text{x}}_{i}^{\prime })-\bar{y}({\text{x}}_{i}^{\prime }){)}^{2}] & =2{\left(k_{-i}^{*}({\text{x}}_{i}^{\prime })+k_{Z}({\text{x}}_{i}^{\prime })/r_{i}\right)}^{2}\\ & \;+4\left(k_{-i}^{*}({\text{x}}_{i}^{\prime })+k_{Z}({\text{x}}_{i}^{\prime })/r_{i}\right)(m_{-i}^{*}({\text{x}}_{i}^{\prime })-\bar{y}({\text{x}}_{i}^{\prime }){)}^{2}. \end{array}$$

We extend ES-LOO to be defined over the full input space by modelling it as a GP with training data ${\text{D}}_{E}=\left\{{\text{X}}^{\prime },{\text{y}}_{E}\right\}$ where ${\text{y}}_{E}=(E_{m}({\text{x}}_{1}^{\prime }),\ldots ,E_{m}({\text{x}}_{n}^{\prime }){)}^{T}$ . This GP, denoted by $Z_{E}(\text{x})$ , has predictive mean and variance given by $m_{E}^{*}(\text{x})$ and $k_{E}^{*}(\text{x})$ , respectively. Due to training a second independent GP, this becomes the most computationally expensive step in the algorithm. When working with very computationally expensive models with limited training data, this additional cost is insignificant. Note that the log of the ES-LOO error is emulated due to ${\text{y}}_{E}$ being positive. Instead of finding the maximum of $m_{E}^{*}(\text{x})$ to propose a new design point, EI (as seen in Bayesian optimization literature [17,39,40]) can be used to ensure a maximum trade-off between exploration and exploitation in the design criteria. The EI is then calculated as:


(2.8) $$\begin{array}{r} EI(\text{x})=\left\{ \begin{array}{ll} \left(m_{E}^{*}(\text{x})-\text{max}({\text{y}}_{E})\right)\Phi (u)+\sqrt{k_{E}^{*}(\text{x})}\phi (u) & \text{if}\;k_{E}^{*}(\text{x})>0\\ 0 & \text{if}\;k_{E}^{*}(\text{x})=0 \end{array}\right., \end{array}$$

where $u=m_{E}^{*}(\text{x})-\text{max}({\text{y}}_{E})/\sqrt{k_{E}^{*}(\text{x})}$ and ϕ(⋅) and Φ(⋅) represent the PDF and CDF of the standard normal distribution, respectively.

To further ensure that there is a sufficient level of exploration in the design, EI is extended to PEI by multiplying the EI by a repulsion function (RF):


(2.9) $$RF(\text{x};\text{X})=\prod_{i=1}^{n}\left[1-\text{Corr}\left(Z_{E}(\text{x}),Z_{E}({\text{x}}_{i}^{\prime })\right)\right],$$

where Corr(⋅,⋅) is the correlation function of $Z_{E}(\cdot )$ . The RF is designed to push potential new points away from existing points in the design. As it is typically not advantageous in most applications to place new design points in the furthest edges and corners of the input space, Mohammadi *et al*. [31] also introduces a set of pseudo points ${\text{X}}_{p}$ into the RF to reduce the chance of points near the boundaries being selected. If it is known that the edges of space give no contribution to the fit of the GP, then extra pseudo points can be included. This is particularly useful when working in higher dimensions when the proportion of edge space increases dramatically. Since the correlation function of $Z_{E}(\cdot )$ is used, this step provides very minor additional computational cost. Finally, a lower bound $\theta_{E}=\sqrt{-0.5/\text{log}(10^{-8})}$ is placed on the correlation length parameters of the GP $Z_{E}$ to ensure that the length scales do not become too small. See [31] for a full discussion on this choice.

Finally, the ESL criteria for a new point is given as:


(2.10) $$\begin{array}{lcr} & {\text{ESL}}_{m}(\text{x})=PEI(\text{x})=EI(\text{x})\cdot RF(\text{x};\text{X}\cup {\text{X}}_{p}). & \end{array}$$

#### (ii) Replicate—calculating ${\text{ESL}}_{\tau }(\text{x})$

We now adapt the ES-LOO criteria from [31] to calculate ${\text{ESL}}_{\tau }(\text{x})$ required for selecting the best existing design point at which to run another replicate. When calculating the ES-LOO for a replicate, we do not know the true value of $\tau^{2}({\text{x}}_{i}^{\prime })$ at each design point and the sample variance is not a good substitute for the true intrinsic variance. The variance measures the spread of data, which inherently requires more information to estimate accurately. First, the calculation of the sample variance requires the sample mean, which is itself an estimate. Second, the squared differences involved in the calculation disproportionately amplify the impact of extreme values, which are more likely to skew results in small samples. This is the main reason why it is not incorporated into the methodology used in hetGP and hence why we cannot use it for design purposes.

We instead replace the true variance with the predicted variance from hetGP. The downside to this is when the estimate of the intrinsic variance is poor in addition to the LOO error being small, we have a poor estimate of the stochastic variance that is not influential to the fit of the GP. This is typically when there are fewer or no replicates at these data points. Since we cannot include any error on the intrinsic variance estimate, this would then imply that no more future replicates in this area are required. In these scenarios, we find that the ES-LOO calculated on the mean response tends to be larger due to the number of replicates causing the influence on the mean to be increased. This hence places more new points in the local area and improves the estimate of the intrinsic variance, and consequently then increases the number of replicates.

To calculate ${\text{ESL}}_{\tau }(\text{x})$ we fit an initial hetGP Z with training data D={X,y} , predicted mean $m^{*}$ and predicted variance $k^{*}=\tau^{2}+k_{Z}$ . On this GP, we perform a LOO-CV by removing each individual point in X in turn. Since we are now interested in the influence of each replicate on the GP, we perform the LOO-CV with respect to each individual point ${\text{x}}_{ij}$ , which includes all $j=1,\ldots ,r_{i}$ replicates at input ${\text{x}}_{i}^{\prime }$ . The resulting LOO GP is defined as $Z_{-ij}(\text{x})$ with predicted mean $m_{-ij}^{*}(\text{x})$ and predicted variance $k_{-ij}^{*}(\text{x})=\tau_{-ij}^{2}(\text{x})+k_{Z,-ij}^{2}(\text{x})$ . Similarly to the previous section, the hyperparameters from Z(x) are used in $Z_{-ij}(\text{x})$ to minimize computational cost. The LOO-CV error is taken to be the difference between the LOO predicted intrinsic variance, and the true intrinsic variance. Since we do not know the true intrinsic variance and the sample variance is not a suitable approximation, we use the predicted intrinsic variance $\tau^{2}(\text{x})$ from the GP. The error at each data point ${\text{x}}_{ij}$ is hence the difference between the LOO predicted intrinsic variance for ${\text{x}}_{ij}$ and the predicted intrinsic variance using the full GP Z :


(2.11) $$\begin{array}{lcr} & e({\text{x}}_{ij})=|\tau_{-ij}^{2}({\text{x}}_{ij})-\tau^{2}({\text{x}}_{i}^{\prime })|. & \end{array}$$

To account for the prediction uncertainty in fitting the GP Z across all replicates and to calculate the LOO error at each unique input ${\text{x}}_{i}^{\prime }$ , we fit a GP $Z_{e}(\text{x})$ to $\tau_{-ij}^{2}(\text{x})-\tau^{2}(\text{x})$ such that


(2.12) $$\begin{array}{lcr} & Z_{e}(\text{x})∼N(m_{e}^{*}(\text{x}),k_{e}^{*}(\text{x})), & \end{array}$$

where $m_{e}^{*}$ is the predicted mean and $k_{e}^{*}$ is the predicted variance. By standardizing this expression, we have the following result:


(2.13) $$\frac{Z_{e}({\text{x}}_{i}^{\prime })-m_{e}^{*}({\text{x}}_{i}^{\prime })}{\sqrt{k_{e}^{*}({\text{x}}_{i}^{\prime })}}∼N\left(0,1\right).$$

By taking the square of the left-hand side, this quantity has a chi-squared distribution with 1 d.f.:


(2.14) $${\left(\frac{Z_{e}({\text{x}}_{i}^{\prime })-m_{e}^{*}({\text{x}}_{i}^{\prime })}{\sqrt{k_{e}^{*}({\text{x}}_{i}^{\prime })}}\right)}^{2}∼\chi^{2}.$$

The expectation is given as follows:


(2.15) $$\begin{array}{lcr} & E\left[{\left(\frac{Z_{e}({\text{x}}_{i}^{\prime })-m_{e}^{*}({\text{x}}_{i}^{\prime })}{\sqrt{k_{e}^{*}({\text{x}}_{i}^{\prime })}}\right)}^{2}\right]=1, & \end{array}$$

where some rearranging gives us the following result for the ESL criterion:


(2.16) $$\begin{array}{lcr} & {\text{ESL}}_{\tau }(\text{x})=E\left[{\left(Z_{e}({\text{x}}_{i}^{\prime })\right)}^{2}\right]=(m_{e}^{*}({\text{x}}_{i}^{\prime }){)}^{2}+k_{e}^{*}({\text{x}}_{i}^{\prime }). & \end{array}$$

We note that we choose not to normalize the ES-LOO result in this case due to not knowing the true error on the intrinsic variance estimate.

### (b) New point or replicate

Given ${\text{ESL}}_{m}(\text{x})$ and ${\text{ESL}}_{\tau }(\text{x})$ , the purpose of the function G in equation (2.1) is to choose between either adding a new point to the current design, or to select an existing point to run a further replicate. The ultimate goal of sequential design is to select the next best point to minimize any uncertainty from fitting the emulator. Hence in the stochastic case, we want to know the overall expected error reduction if either the new point or the replicate is chosen.

#### (i) Maximum entropy and maximum predictive variance

Entropy measures the amount of uncertainty or information contained within a probability distribution. A maximum entropy design [1,12,15] is an approach to strategically select data points that maximize the potential information gained about a complex model: points that maximize the entropy, or uncertainty, of the underlying probability distribution. By doing so, the design avoids sampling bias and ensures that the selected points fully explore the input space and capture all possible variations.

The maximum entropy design criterion can be modified for use as a sequential algorithm [41,42]. The covariance matrix between the training data and the potential new point (either a completely new point or a replicate) can be partitioned into


(2.17) $$\begin{array}{r} \Sigma =\left(\begin{array}{cc} k(\text{X},\text{X})+\tau^{2}(\text{X})I_{N} & k(\text{x},\text{X})\\ k(\text{X},\text{x}) & 1 \end{array}\right) \end{array},$$

where $k(\text{X},\text{X})+\tau^{2}(\text{X})I_{N}$ is the correlation matrix based on the existing N design points only. The cross correlation between the candidate point x (new or replicate) and the existing design points is denoted by the vector k(x,X) . To gain as much information as possible, the maximum entropy approach selects a new point that maximizes the determinant of the prior covariance Σ . Jin *et al*. [43], showed that if points are selected one at a time sequentially, then the maximum entropy approach becomes equivalent to maximizing the mean squared prediction error. Hence, the sequential maximum entropy criterion reduces to the following for G :


(2.18) $$G(\text{x})=1-k(\text{x},\text{X})(k(\text{X},\text{X})+\tau^{2}(\text{X})I_{n}{)}^{-1}k(\text{X},\text{x})=k^{*}(\text{x}).$$

The predictive variance is a well-known criterion for adaptive design methods [43,44] and often a new point is chosen where the predictive variance from the GP is largest. The criterion is typically space filling since the uncertainty increases away from existing data points. A disadvantage of this is that the uncertainty is high around the boundaries of the input space where there are typically fewer points. This is not advantageous in many circumstances where areas of interest do not lie near the boundaries, and hence the criterion becomes a significant issue in higher dimensions where the boundary areas increase substantially. Since we are only using this criterion to select between two previously selected points (new point or replicate) this should not be an issue: the ES-LOO criterion will already ensure that we are not choosing uninformative new points close to boundaries, hence we are confident that this choice for G will scale to high dimensions.

#### (ii) Integrated mean squared prediction error

An alternative to selecting points at maximum predictive variance is to select points based on the IMSPE [1,30]:


(2.19) $$\begin{array}{lcr} & G(\text{x})=\text{IMSPE}(\text{x})=\int_{\chi }k^{*}(\text{x})\;d\text{x}. & \end{array}$$

Unlike methods that focus on areas of high uncertainty, IMSPE aims to reduce prediction error across the entire input space, leading to a more globally accurate GP. IMSPE is therefore able to balance between exploring new areas of the input space and refining predictions in already sampled regions.

Evaluating IMSPE requires calculating the predictive variance over the entire input space for each candidate point, which becomes computationally expensive, especially in high-dimensional problems. Since we only have two candidate points when choosing between either the new point or the replicate, the computational cost is greatly reduced, however, estimating across the full input space is still expensive.

The effectiveness of IMSPE relies on the accuracy of the underlying GP. If the GP is poorly specified, or has a poor fit, then the selected points may not effectively reduce prediction error. This is particularly true in cases of heteroscedastic noise, where accurately capturing the varying noise levels across the input space is crucial. Poor estimation of the intrinsic noise can lead to suboptimal designs where IMSPE often places more points near the boundaries where the variance is larger. This tendency can limit the method’s effectiveness in regions with high variance.

#### (iii) Random

The last criterion we will consider is to simply select either a new point or replicate at random. In line with equation (2.1), we define G to be equivalent to drawing a sample from a uniform (0,1) distribution. Either a new point or replicate is chosen based on which draw is larger. Although selecting randomly is very cheap, it will not always give the optimum.

### (c) Many replicates

As mentioned previously, when there are enough replicates at each design point [20], then the sample mean and sample variance can be used as approximations to the true mean response and the intrinsic variance, respectively. In this case, we can fit distinct GPs for both of these quantities. Since each of these GPs are treated to be deterministic (we treat the intrinsic variance GP to have constant variance), we can therefore use the ES-LOO sequential design for calculating ${\text{ESL}}_{m}$ in §2a(i) for both the new point (mean) and the replication (variance). For the calculation of ${\text{ESL}}_{\tau }$ the sample mean ( $\bar{y}$ ) is replaced with the sample variance ( $s^{2}$ ). Similarly to §2a(ii), since we do not need to optimize ${\text{ESL}}_{\tau }$ across the full input space, the criteria stop with equation (2.7), and we need only calculate the ES-LOO for each existing design point.

The previous section outlined how to choose between selecting either a new point or a replicate. This can also be used when there are many replicates (following equation 2.1), however, if this is the case, then it can also be assumed that the model is cheap enough for further replicates to be evaluated. We can therefore assume a new point is generated both from calculating ${\text{ESL}}_{m}(\text{x})$ and ${\text{ESL}}_{\tau }(\text{x})$ and subsequent replicates are also performed. $PEI_{m}(\text{x})$ is still used to select new points that will improve the mean response, while $PEI_{\tau }(\text{x})$ now selects new points that will help to improve the intrinsic variance. The ES-LOO for ${\text{ESL}}_{\tau }$ is calculated as described above and PEI is then further used to select a point across the full input space. This method can also be used for cases where the cost of running the model is the same regardless of how many replicates we want to perform.

### (d) Batch design

The sequential design approach can also be run in batch mode to include q new points at each iteration. To do this, we select the best q new design points as well as the best q replicates and then choose the best q overall points from this selection.

To select the best q new points, similarly to [31], we adapt the RF used to calculate the PEI. This then stops the same point being chosen twice. Hence, we can update the RF for a batch of q runs as follows:


(2.20) $$RF(\text{x};\text{X}\cup {\text{x}}_{n+1}\cup \ldots \cup {\text{x}}_{n+q-1})\;\;\;\;\;\;\;\;=\prod_{i=1}^{n+q-1}[1-\text{Corr}(Z_{E}(\text{x}),Z_{E}({\text{x}}_{i}))],$$

for $\text{X}\;=({\text{x}}_{1},\ldots ,{\text{x}}_{n})$ .

To select the best q replicates is more challenging as we must make sure to consider adding a single point for more than one replication. In this respect, the decision is given to the user on whether they want to include multiple replicates at the same point. If no multiple replications are taken, then we can simply select the q highest values of $E[(Z_{e}({\text{x}}_{i}){)}^{2}]$ . However, when it comes to deciding how many replicates of each point to take, then there is no clear answer. It may also be more beneficial to include replicates of the new points selected rather than the existing points. Hence, we would suggest prioritizing points with either the highest values of $E[(Z_{e}({\text{x}}_{i}){)}^{2}]$ or points with the fewest replicates.

## 3. Examples

In this section, we present three stochastic examples with different numbers of inputs to explore how the method behaves in both low and high dimensions. The first example is a simple two-dimensional toy example, the second is an ABM that simulates the spread of an infectious disease in a semi-closed population, and the last example simulates the spread of COVID-19 to predict the number of infected, hospitalized, recovered and deceased patients.

The performance of our method is compared against several alternatives. We first test the stochastic ES-LOO method with three different versions of G for choosing either a new point or a replicate (as detailed in §2b): an entropy selection (ES-LOO + Entropy), an IMSPE selection (ES-LOO + IMSPE) and a random selection (ES-LOO + Random). Finally, we make a comparison with the existing hetGP with IMSPE approach from [26] (hetGP IMSPE).

To test the accuracy of the different design approaches, we use both the normalized root mean square error (NRMSE) and the score criteria. Given a test set $D_{t}=\{{\text{X}}_{t},f({\text{X}}_{t}){\}}_{t=1}^{t=N}$ , where $D_{t}^{\prime }=\{{\text{X}}_{t}^{\prime },f({\text{X}}_{t}^{\prime }){\}}_{t=1}^{t=n}$ , for n≪N is the set of unique values in $D_{t}$ , the NRMSE is given by:


(3.1) $$\begin{array}{lcr} & \begin{array}{rl} {\text{NRMSE}}_{m}({\text{x}}_{t}^{\prime }) & =\frac{\sqrt{(\sum_{t=1}^{n}(m^{*}({\text{x}}_{t}^{\prime })-\bar{y}({\text{x}}_{t}^{\prime }){)}^{2})/n}}{\underset{{\text{x}}_{t}^{\prime }\in D_{t}^{\prime }}{\max\;}\bar{y}({\text{x}}_{t}^{\prime })-\underset{{\text{x}}_{t}^{\prime }\in D_{t}^{\prime }}{\min\;}\bar{y}({\text{x}}_{t}^{\prime })},\\ {\text{NRMSE}}_{\tau }({\text{x}}_{t}^{\prime }) & =\frac{\sqrt{(\sum_{t=1}^{n}(k^{*}({\text{x}}_{t}^{\prime })-s^{2}({\text{x}}_{t}^{\prime }){)}^{2})/n}}{\underset{{\text{x}}_{t}^{\prime }\in D_{t}^{\prime }}{\max\;}s^{2}({\text{x}}_{t}^{\prime })-\underset{{\text{x}}_{t}^{\prime }\in D_{t}^{\prime }}{\min\;}s^{2}({\text{x}}_{t}^{\prime })}, \end{array} & \end{array}$$

where ${\text{NRMSE}}_{m}$ is the NRMSE evaluated on the mean response, ${\text{NRMSE}}_{\tau }$ is the NRMSE evaluated on the variance output and where $m^{*}({\text{x}}_{t}^{\prime })$ and $k^{*}({\text{x}}_{t}^{\prime })$ are the expected mean and variance output of the GP at input ${\text{x}}_{t}^{\prime }$ . $\bar{y}({\text{x}}_{t}^{\prime })$ and $s^{2}({\text{x}}_{t}^{\prime })$ are the sample mean and variance at input ${\text{x}}_{t}^{\prime }$ over $r_{t}$ replicates. The score is given by:


(3.2) $$\begin{array}{lcr} & \text{Score}({\text{x}}_{t}^{\prime })=\frac{1}{n}\sum_{t=1}^{n}(-((m^{*}({\text{x}}_{t}^{\prime })-\bar{y}({\text{x}}_{t}^{\prime }))/\sqrt{k^{*}({\text{x}}_{t}^{\prime })}{)}^{2}-\text{log}(k^{*}({\text{x}}_{t}^{\prime }))). & \end{array}$$

Lower values for NRMSE are preferred, while higher values for the score criteria indicate an improved model. The score can be used for stochastic examples since it considers both the mean response and the variance.

We select n=10000 test points for the two-dimensional example in a random Latin hypercube across the input space, with 250 replicates at each point to calculate both the sample mean and sample variance. For the ABM example, we select n=250 test points with 100 replicates, and for the COVID-19 example, we select n=100 test points with 100 replicates.

### (a) Example I

The mean response of the two-dimensional toy example is given by the following function:


(3.3) $$F(x_{1},x_{2})=4(x_{2}+x_{1}^{2}+x_{2}^{2}+\text{sin}(4\pi x_{1}x_{2})-\sqrt{2}),$$

where the noise function is given as:


(3.4) $$\begin{array}{lcr} & \tau (x_{1},x_{2})=a\cdot (0.25\cdot x_{1}+0.75\cdot x_{2}). & \end{array}$$

We consider two cases for the noise function: reduced noise where a=2 , and large noise where a=10 . We have chosen to perform the IMSPE version with a replicate look ahead of two steps since this is the default option in the hetGP R package. From prior results this is the version that performed the best for the toy example, giving the fairest comparison. Selecting no look ahead steps provided a sequential design with few replicates.

For both the reduced and increased noise examples, we start with an initial maximin Latin hypercube design (LHD) of six points and four replicates at each of these points to give a total of 24 model runs. We then run the stochastic ES-LOO algorithm to add a further 50 points to the design. The algorithm is run for 10 different starting designs (maximin LHDs) and NRMSE scores for the test set are averaged to ensure the initial design does not create any bias.

**Figure 1.**
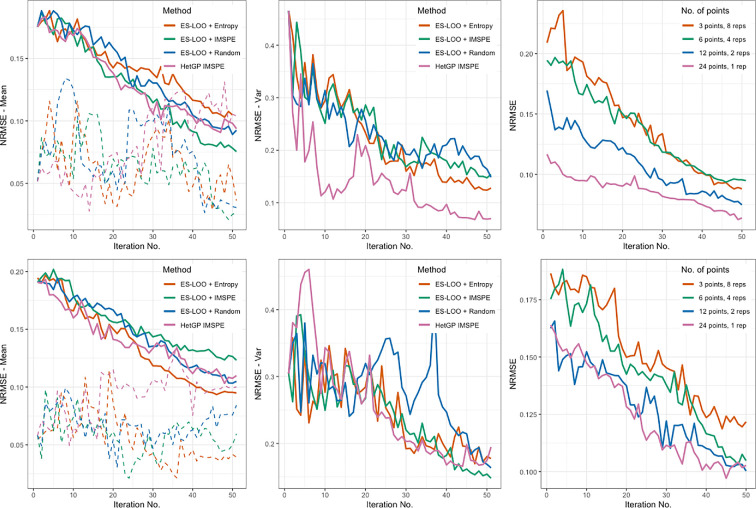
Plots showing the NRMSE scores for the toy example in §3a. Top row: Reduced noise case when a=2 . Bottom row: When a=10 . (Left) NRMSE scores when compared against the predicted mean along with the ranges of ${\text{NRMSE}}_{m}$ results (max − min) over the 10 different LHDs. (Middle) NRMSE scores when compared against the predicted variance. (Right) NRMSE scores for the predicted mean using ES-LOO + Entropy with different initial LHD sizes and numbers of replications.

Figure 1 shows the results where the top row corresponds to the reduced noise case ( a=2 ), and the bottom row corresponds to a=10 . From left to right, the first plot shows ${\text{NRMSE}}_{m}$ and the second plot shows ${\text{NRMSE}}_{\tau }$ . When a=2 , the ${\text{NRMSE}}_{m}$ scores show little difference for all methods, implying each method performs similarly in improving the mean response prediction from the GP. However, for the variance, it is clear that the hetGP IMSPE method performs best. When the noise is increased ( a=10 ), we instead find that the ES-LOO approach with the entropy selection performs the best, with ES-LOO + IMSPE performing the worst. The ${\text{NRMSE}}_{\tau }$ scores show little difference between methods. The dashed lines show the ranges of ${\text{NRMSE}}_{m}$ results (max − min) over the 10 different starting designs. For a=2 , the ranges are similar for all methods, with the exception that hetGP IMSPE increases with the number of iterations. The hetGP IMSPE method also has the largest range for a=10 , with ES-LOO + Entropy having the smallest range. The ranges for ${\text{NRMSE}}_{\tau }$ were very similar for each method (and so not included in figure 1).

The two plots on the right in figure 1 show ${\text{NRMSE}}_{m}$ when both the sizes of the initial LHDs and the number of replicates at each initial points vary. The ES-LOO + Entropy algorithm is run for 50 iterations with designs consisting of three points and eight replications, six points and four replications, 12 points and two replications, 24 points and no replications, each having a total of 24 points. Results are averaged over 10 different maximin LHDs. For a=2 , the NRMSE scores for the initial designs with three and six points are very similar, indicating that the increase in replications has little effect. The other two starting designs begin with a lower NRMSE: when the intrinsic variance is lower, including more single points and fewer replicates is more beneficial to the accuracy of the mean prediction. The NRMSE decreases at a lower rate since many of the iterations add in replicates to learn about the intrinsic variance and a good estimate of the mean has already been achieved. The results for a=10 are very similar, however, there is a lot more variability due to the increased variance.

**Figure 2.**
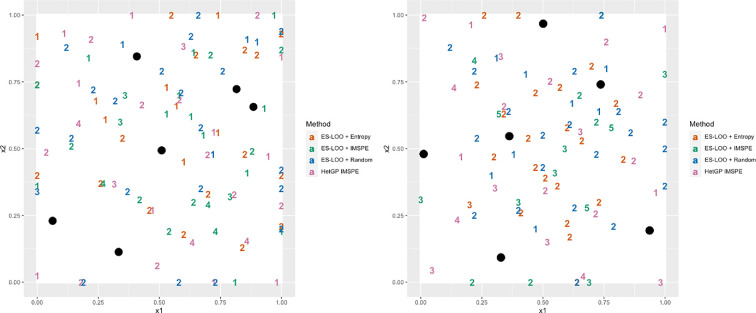
Plots showing the number of replicates at each design point after performing 50 iterations of a sequential design method for the toy example in §3a. (Left) Reduced noise case when a=2 . (Right) When *a* = 10. Black points represent the initial design, each with four replicates.

The numbers of new points added versus the number of replicates added were similar across all methods, with the ES-LOO methods favouring replicates slightly more than the hetGP IMSPE approach. The total numbers of replicates after performing the 50 iterations of each sequential design method are presented in figure 2 (left: a=2 , right: a=10 ). The starting designs are given in the solid black dots, where four replicates were run for each. Overall, all methods place new and replicated points in similar patterns. When a=2 , there are more single points for all methods and the majority of points show a high level of exploration. The exception is that both ES-LOO + Entropy and ES-LOO + IMSPE methods did not place any further runs in the bottom left-hand corner, indicating that this area of input space has little information and low variance. This agrees with the true model. When a=10 , we now note that the ES-LOO + Entropy method places the fewest points near the boundaries of space, and are more clustered to the centre. There are mostly two replicates at each point, indicating that this method prefers to learn the increased intrinsic variance by placing a higher number of points closer together. Only the hetGP IMSPE and ES-LOO + Random selection methods are shown to have single points with no replicates in this example. The least number of replicates were chosen by the ES-LOO + Random method, indicating the choice of G affects the number of replicates.

### (b) Example II: Agent-based model

Our second example is the epiDEM Travel and Control ABM run in NetLogo [45], which is an extension to epiDEM (Epidemiology: Understanding Disease Dynamics and Emergence through Modelling) to include features such as travel, isolation, quarantine, inoculation and links between individuals.

Individuals are divided into two groups which are then separated by a border and allowed to move randomly across the entire space. Each individual has a 5% chance of being initialized as infected. When a person contacts an infected individual, they may contract the illness. Based on user-defined inputs, infected individuals may self-isolate, go to a hospital, be force-quarantined or continue moving. After a set recovery time, infected individuals have a chance of recovering and becoming permanently immune. There is an additional agent that represents a health official or ambulance, which locates and transports infected individuals to the hospital.

There are nine controllable variables in the model, with an additional three switch variables that are kept constant throughout. The inputs include the total number of initial individuals, the chance of infection and recovery, the average recovery time and average isolation tendency, the average tendency of the individual choosing to go to hospital, the probability of an individual being vaccinated (and hence immune), how mobile each individual is and the probability that each individual will travel at each time step. The model tracks changes in cumulative infections and recoveries, along with the average number of secondary infections. The reproduction number $R_{0}$ estimates the virus’s spread, considering multiple infected individuals and user-defined variables. At the simulation’s end, $R_{0}$ reflects the average secondary infections per case and determines if an epidemic occurred. It is calculated as:


(3.5) $$R_{0}=\frac{N\;\text{log}(S(0)/S(t))}{N-S(t)},$$

where N is the population size, S(0) is the initial number of susceptibles and S(t) is the number of susceptibles at time t .

**Figure 3.**
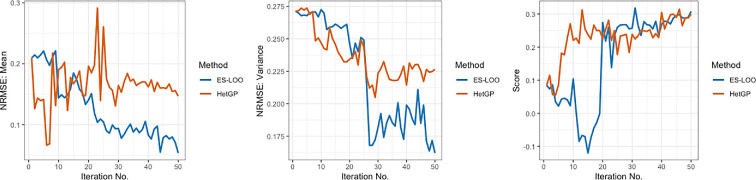
Plots showing the NRMSE and score results from applying 50 iterations of the ES-LOO (blue) and hetGP with IMSPE (orange) sequential design methods to the epiDEM ABM. (Left) NRMSE for the mean, (middle) NRMSE for the variance and (right) NRMSE for the score.

We model $R_{0}$ at the end of the simulation given the nine inputs. We initially generate a Latin hypercube with 50 points in nine-dimensional space, which is run through the ABM and replicated five times at each design point. The stochastic ES-LOO algorithm with entropy choice for G is compared against the hetGP with IMSPE approach, where both methods are run for 50 iterations. The NRMSE for both the mean response and variance, along with the score metrics, are plotted in figure 3. In higher dimensions, the ${\text{NRMSE}}_{m}$ score for the ES-LOO approach consistently reduces as the number of iterations of the sequential design increases. This is not the case for hetGP IMSPE, where the ${\text{NRMSE}}_{m}$ remains constant on average. Both methods reduce in ${\text{NRMSE}}_{\tau }$ . In this example, the hetGP approach favours either adding new points close in Euclidean distance or adding replicates to the design, both of which have a greater influence in the accuracy of the intrinsic variance estimate. The ES-LOO approach was shown to add additional new points to the design, where very few replicates were initially included. The large drop-in ${\text{NRMSE}}_{\tau }$ indicates when replicates were introduced to the design.

For stochastic sequential designs, we often see places where the NRMSE is shown to greatly fluctuate. This happens when there are few (or no) replicates at certain points and the intrinsic variance is poorly estimated. As soon as we include more replicates at these points (or new points in the close neighbourhood), we learn more about the true intrinsic variance. These large fluctuations are seen in both design methods.

### (c) Example III: EERA COVID-19 model

Our last example is the Epidemiology, Economics and Risk Assessment (EERA) model [33], which is designed to simulate the spread of the COVID-19 pandemic in Scotland. This model compartmentalizes the population into either susceptible, exposed, three different levels of infection, hospitalized, recovered and died (SEI3HRD). Anyone who becomes exposed will become pre-clinically infectious and from there some will become either asymptomatic or symptomatic. The asymptomatics stay infectious for a long period of time but all will recover whereas a portion of symptomatics will either recover, be hospitalized or die. Those hospitalized will either recover or die. This flow is shown in figure 1, page 2 of [46]. Individuals move between the aforementioned compartments through the specification of probabilities. These probabilities depend on parameters that are specified either by the user or through Bayesian inference using real-world data and prior specifications. The prediction mode of the model simulates the pandemic from day 1 to a specified end day and simulates the number of individuals in seven different age groups, as well as healthcare workers.

There are a total of 14 unknown parameters in the model. A previous study on the model performed a variance-based sensitivity analysis [46] to find the main effects and interactions of all 14 parameters. The results showed that there were five parameters that had a significant effect on the model. These parameters were probability of infection, proportion of population observing social distancing, proportion of normal contact made by people self-isolating, age-dependent probability of developing symptoms and mean asymptomatic period in days. The range of these parameters, along with detailed descriptions of all parameters, are given in table 1 in [46].

**Figure 4.**
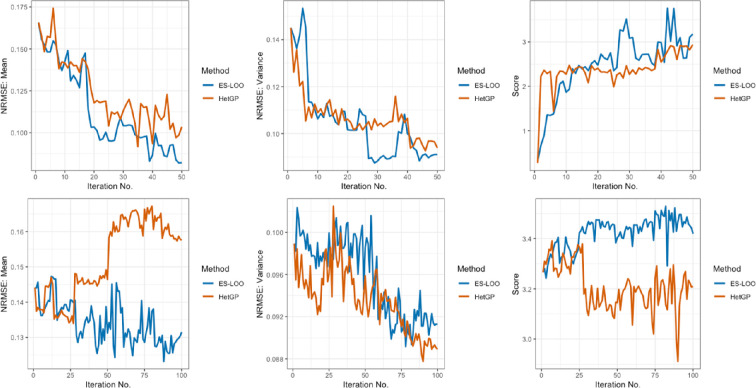
Plots showing the NRMSE and score results from applying 50 five-dimensional and 100 14-dimensional iterations of the ES-LOO (blue) and hetGP with IMSPE (orange) sequential design methods to the COVID-19 EERA model. (Top) five-dimensional model. Bottom: 14-dimensional model. (Left) NRMSE for the mean. (Middle) NRMSE for the variance. (Right) NRMSE for the score.

We apply both the ES-LOOO + Entropy and hetGP IMSPE design methods to the full 14-dimensional (14D) model as well as the reduced five-dimensional model. In the five-dimensional case, all other parameters are held at default values. The output is the number of deaths in patients aged over 70. The model is run for 200 time steps (days) (as in [46]), and the total number of deaths is recorded in this age group. We initially generate a Latin hypercube with 20 points for the five-dimensional model and 50 points for the 14-dimensional model. Both models are run for five replicates at each point. We then run 50 sequential design iterations for the five-dimensional model and 100 iterations for the 14-dimensional model. The results for both examples (top: five-dimensional, bottom: 14-dimensional) and both methods (blue: ES-LOO, orange: hetGP) are given in figure 4.

From left to right the plots show the reduction in NRMSE against the mean, the NRMSE against the true intrinsic variance and the score. For the five-dimensional model, we can see that both methods successfully select points that reduce the NRMSE and therefore increase the accuracy of the GP. This is echoed in their similar increase in score values. Their performance across all criteria is very similar, with hetGP choosing slightly more replicates than ES-LOO (24 versus 19).

When we increase the dimension to 14, the performance of both methods decreases. The ${\text{NRMSE}}_{m}$ score reduces slightly for ES-LOO, but actually increases for the hetGP approach. Again, high fluctuations are seen for both methods, which is a common occurrence for heteroscedastic sequential design methods, particularly when more replications are included. Similar observations can be seen for ${\text{NRMSE}}_{\tau }$ , where it reduces overall for both methods, with a high level of fluctuation. The score increases for ES-LOO, but reduces slightly for hetGP. The numbers of replicates chosen are similar to the five-dimensional model with 58 for hetGP and 39 for ES-LOO.

## 4. Conclusion

Uncertainty quantification for healthcare and biological applications is a topic of great interest due to the increased use of computer models and digital twins. One of the greatest difficulties is that these biological models have different underlying structures and assumptions to equivalent models in engineering and physical science. This results in the need for increased research to adapt these methods to be applicable to a wider range of problems. These models tend to have a very high and intricate intrinsic variance structure due to the many random components involved. There is limited literature on sequential design methods for stochastic models, due to the complex choice of either adding a new point or a replicate at each iteration. The current leading method was developed by Binois *et al*. [26], who focused on minimizing the IMSPE when selecting either a new point or a replicate. Due to the poor scalability to higher dimensions of this method, we have investigated alternative approaches and their suitability for healthcare applications.

We have presented our new approach for sequential design for stochastic models, with motivation for epidemiological applications. The methodology is an adaptation of the deterministic version developed by Mohammadi *et al.* [31] whereby we simultaneously select the next best new design point and next best replication. Once we have made these selections, we then apply a separate criterion to choose between adding a new point and adding a replicate to the final design. The three criteria we considered are maximum entropy (maximum predictive variance), maximum IMSPE and a random selection. Since we are assuming that the computer models tend to be expensive to run, we also assume that we typically do not have many replicates at each of the design points. Due to this reason, we use the heteroscedastic GP, known as hetGP, to fit the majority of the GPs with stochastic outputs. We have given details regarding an extension to a batch design, as well as alterations that can be made if the stochastic model is cheap to run and we can therefore obtain many replicates at each design point. Results from a toy example showed that the ES-LOO method combined with an entropy selection performed the best when the intrinsic variance was large. All methods (including hetGP IMSPE) performed equally at reducing the uncertainty on the mean response estimate when the noise is low. We applied the methodology to an ABM for infectious diseases and a COVID-19 model that simulates the spread of the virus in Scotland.

Results showed that the ES-LOO sequential design performed better than the hetGP IMSPE approach in higher dimensions. For the nine-dimensional ABM, the NRMSE for both the mean and variance were shown to decrease for the ES-LOO approach while only the NRMSE for the variance reduced for the hetGP IMSPE approach. This is reflected in the results for the 14-dimensional COVID model with the exception that the NRMSE for the mean response actually increased in the hetGP IMSPE case. While the ES-LOO design effectively improves both the mean and variance predictions, the hetGP IMSPE design only improves variance predictions. This is due to both an increased number of replicates and a lack of exploration with the majority of additional new points being placed close to Euclidean distance. In higher dimensions, the key feature of the ES-LOO design method is that it remains space filling: the algorithm tends to initially include more new points, while replications are performed in latter iterations. This initial exploration step has a greater influence on the fit of the emulator, causing the NRMSE on the mean response to reduce.

There are areas where the methodology could be improved. The first of these is to better define the batch design approach since it is challenging to successfully quantify the number of optimum replicates to perform at each chosen new point or replicate. The second is to consider whether a look ahead over the replication approach is possible. This will help in guiding the batch design since we will be able to consider future possible combinations of new points and replications when making the current selection. We would be able to consider the influence of future potential replicates during the selection of new design points. This is a tool that influences the results of the IMSPE design method in [26], and so has great potential with the ES-LOO methodology.

## Data Availability

Supplementary material is available online [47].
